# Health Disparities among adults cared for at an urban cystic fibrosis program

**DOI:** 10.1186/s13023-021-01965-4

**Published:** 2021-07-31

**Authors:** Emily DiMango, Kaitlyn Simpson, Elizabeth Menten, Claire Keating, Weijia Fan, Cheng-Shiun Leu

**Affiliations:** 1 Columbia University Irving Medical Center, 622 West 168th Street, New York, NY 10032 USA; 2 Columbia University Mailman School of Public Health, 722 West 168th Street, New York, NY 10032 USA

**Keywords:** Cystic fibrosis, Disparities, Health outcomes, Rare disease

## Abstract

**Background:**

Evidence is conflicting regarding differential health outcomes in racial and ethnic minorities with cystic fibrosis (CF), a rare genetic disease affecting approximately 28,000 Americans. We performed a cross-sectional analysis of health outcomes in Black/Latinx patients compared with non-Hispanic Caucasian patients cared for in a CF center in New York City. Adult patients enrolled in the CF Foundation Patient Registry at the Columbia University Adult CF Program and seen at least once during 2019 were included. Health metrics were compared between Black/Latinx and non-Hispanic Caucasian patients.

**Results:**

262 patients were eligible. 39 patients (15%) identified as Black/Latinx or non-Hispanic Caucasian. Descriptive statistics are reported with mean (standard deviation). Current age was 35.9 (13.3) years for non-Hispanic Caucasian and 32.0 (9.3) years for Black/Latinx patients (*p* = 0.087). Age of diagnosis did not differ between groups; 9.56 (15.96) years versus 11.59 (15.8) years for non-Hispanic Caucasian versus Black/Latinx respectively (*p* = 0.464). Pulmonary function, measured as mean forced expiratory volume in one second (FEV1) was 70.6 (22.5) percent predicted in non-Hispanic Caucasian versus 59.50 (27.9) percent predicted in Black/Latinx patients (*p* = 0.010). Number of visits to the CF clinic were similar between groups. When controlled for age, gender, co-morbidities, median income, and insurance status, there was a continued association between minority status and lower FEV1.

**Conclusions:**

Minorities with CF have significantly lower pulmonary function, the major marker of survival, than non-Hispanic Caucasians, even when controlled for a variety of demographic and socioeconomic factors that are known to affect health status in CF. Significant health disparities based on race and ethnicity exist at a single CF center in New York City, despite apparent similarities in access to guideline based care at an accredited CF Center. This data confirms the importance of design of culturally appropriate preventative and management strategies to better understand how to direct interventions to this vulnerable population with a rare disease.

## Introduction

Cystic fibrosis is an autosomal recessive disease affecting nearly 28,000 Americans and is the most common lethal genetic disease among white people [1, 2]. The disease primarily affects the gastrointestinal and respiratory tracts, ultimately leading to respiratory failure. Forced expiratory volume in one second (FEV1) is a measure of lung function and is the most sensitive indicator of lung disease severity [3]. There have been tremendous improvements in survival for individuals with cystic fibrosis (CF) over the past several decades, with median survival increasing from 27 years in 1986 to 46 years in 2019 [2]. The recent development of cystic fibrosis transmembrane regulator (CFTR) modulators is believed to have the potential to increase life expectancy significantly, since these compounds have been shown to modify disease progression in the lungs [4, 5]. Despite this, certain subpopulations may still remain at risk for worse clinical outcomes.

Although CF is predominantly a disease of Caucasians, the proportion of affected patients who are racial and ethnic minorities is increasing, following demographic trends in general in the US [2, 6]. For example, the number of patients with CF who are of Hispanic ethnicity comprised 9.4% of the CF population in 2019 compared to 6.1% in 2004 [2]. Black patients made up 4.7% of the CF population in 2019 compared with 3.9% in 2004. Minority patients are a vulnerable population with high risk for worse health outcomes [7]. Patients with CF differ from those with many other chronic diseases in that CF is a rare disease, the genetic basis of CF is well defined, and most patients with CF receive guideline directed specialty care at accredited CF care centers.

While poorer health outcomes have been reported in individuals with CF and lower socioeconomic status (SES) [8], there is conflicting evidence regarding health outcomes in racial and ethnic minorities with CF, with apparent geographic differences playing a role in disparities (6–9). Hispanic patients with CF in California were found to have a 2.8 higher mortality rate than non-Hispanic patients with CF, however Hispanic patients in Texas had similar outcomes to those of non- Hispanics [9]. Rho reported that Hispanics with CF had a 1.4 year shorter life span than non-Hispanics and a 1.27 higher risk of death, with disparities most affecting those living in specific regions of the US [10]. This data highlights the fact that there are regional differences in outcomes of minority patients with CF. Defining and understanding health disparities in this rare disease is particularly critical in light of recently approved treatments that have the potential to significantly prolong life.

As a CF center in New York City with a relatively large minority population, we sought to determine if racial and ethnic disparities in health outcomes exist and to further understand possible causes for any existing disparities.

## Methods

The study was approved by the Columbia University Institutional Review Board. This is a retrospective cross-sectional analysis of adult patients enrolled in the Cystic Fibrosis Foundation (CFF) Patient Registry at the Columbia University Adult CF Program and seen at least once during 2019. 96% of patients followed at the Center have provided signed consent for inclusion in the Registry. Patients who underwent lung transplantation at any time prior to or during the year of analysis were excluded. The Registry collects basic demographic, diagnostic, clinical and outcome data for individuals each time they are seen in the outpatient or inpatient setting. Ethnicity is collected by self-report. Genetic mutations are classified by presence of one, two or no F508*del* mutations; F508*del* is the most common mutation in CF with approximately 45% of patients homozygous and 45% heterozygous [2]. F508*del* is considered a severe mutation in CF and is associated with worse outcomes.

The primary predictor of interest was race/ethnicity, categorized as Black/Latinx (BLx) or non-Hispanic Caucasian (NHC). Clinical and biological factors including age, body mass index (BMI), forced expiratory volume in one second (FEV1), F508*del* mutation, presence of diabetes, presence of *Pseudomonas aeruginosa* in the lungs and age at diagnosis were compared between BLx and NHC patients. Mean FEV1 and BMI for the year were calculated as the average of the maximal value reported for each quarter. *Pseudomonas* infection refers to presence during the reported year. Other predictors of outcomes were identified based on social factors most likely to mediate or confound any relationship between race/ethnicity and health outcomes; specifically Medicaid insurance status and median household income estimated by zip code of residence. Markers of medical access such as number of outpatient health related encounters to the CF clinic, hospitalizations, or use of intravenous antibiotics for treatment of CF pulmonary exacerbations over the year were tabulated.

### Statistical analysis

Descriptive statistics were generated and compared by minority status for samples, characteristics, and outcomes of interest. We reported mean (standard deviation) and frequency (percentage) for continuous and categorical variables respectively. Comparison by minority status was conducted using t-test for continuous variables and Chi-squared test for categorical variables. Linear regression analysis was applied to examine whether minority status was associated with FEV1. The analysis was done on the whole sample and by genotype (presence of F508*del* mutation). Patients’ gender, age, age at diagnosis, presence of diabetes, median household income based on zip code of residence, and insurance type (private/Medicare vs. public (Medicaid)) were also included in the model to adjust for potential confounding. We report the unadjusted and adjusted regression coefficient of minority status (β) as well as its corresponding 95% confidence interval and *p *value. We declare the association as statistically significant if *p* ≤ 0.05. Findings with *p* > 0.05 but ≤ 0.10 supported a trend toward statistically significant.

## Results

262 eligible patients were enrolled in the Cystic Fibrosis Registry and had at least one visit during 2019. 39 patients (15%) identified as Black (n = 7) or Hispanic/Latinx. Mean current age was 35.9 (13.3) years for NHC and 32.0 (9.3) years for BLx patients with no group difference (*p* = 0.087). Mean age of diagnosis did not differ between groups; 9.56 (15.9) years versus 11.59 (15.8) years, for NHC versus BLx respectively (*p* = 0.46). One hundred and sixteen (52%) of NHC patients were diagnosed before age 2 years compared with eighteen (46%) of BLx patients. BLx patients had a significantly lower median household income based on zip code of residence ($68,485) compared with NHC ($102,492), *p* < 0.001 (Table 1).

**Table 1:** Demographic characteristics and health metrics

	Non-Hispanic Caucasian (n = 223)	Black/LatinX (n = 39)	*p*
Female (n, %)	125 (56.1%)	17 (43.6%)	0.149
Mean current age *(standard deviation SD)	35.9 (13.3))	32.1 (9.3)	0.087
Mean age at diagnosis, years *	9.56 (15.9)	11.59 (15.8)	0.46
Mean sweat chloride *	92.9 (4.5)	90.4 (1.7)	0.87
Mean FEV1 L *	3.01 (7.08)	2.19L (1.07)	0.47
Mean FEV1 (%) *	70.6 (22.5)	59.5 (27.92)	0.010
Mean FVC L *	3.68 (1.13)	3.15 (1.19)	0.008
Mean FVC (% predicted) *	84.17 (18.46)	73.00 (24.69)	0.002
FEV1/FVC *	0.77 (1.34)	0.67 (0.15)	0.65
Mean BMI *	23.02 (4.09)	22.44 (4.20)	0.446
Diabetes (n, %)	56 (25.1)	14 (35.9)	0.160
Pseudomonas aeruginosa (n, %)	138 (62)	25 (64)	0.67
Mean no. encounters	4.64 (0.17)	4.4 (0.38)	0.96
Mean no. Intravenous antibiotic (IV) courses *	0.68 (0.07)	0.74 (0.22)	0.67
Hospitalizations *	0.27 (0.05)	0.86 (0.15)	0.14
Days treated with IV antibiotics *	11.5 (1.3)	12.23 (3.9)	0.92
Median income *	102,494 (36,060)	68,485 (33,159)	< 0.001
Medicaid (n, %)	18 (8.7%)	17 (45.9%)	< 0.001
Medicaid/Medicare (n, %)	39 (17.5%)	19 (48.7%)	< 0.001
Treated with modulators (n, %) in 2019	116 (52%)	13 (38%)	0.78
Eligible for elexacaftor/tezacaftor/ivacaftor	170 (76%)	24 (62%)	0.87

^*^Standard deviation

Mean FEV1 percent predicted was 59.50 (27.9) in BLx patients vs. 70.6 (22.5) in NHC, (β = -11.10, 95% CI = (-19.58, -2.63), *p* = 0.010). Mean BMI, total number of outpatient clinic encounters, care episodes (treatment with intravenous antibiotics), number of hospitalizations, and number of intravenous antibiotic days did not differ between groups (Table 1).

When adjusted for gender, age, age at diagnosis, presence of diabetes, median household income based on zip code of residence and insurance type, the association between minority status and lower FEV1 decreased and trended toward statistical significance (β = -8.38, 95% CI = (-17.51, 0.75), *p* = 0.072).

Table 2 shows unadjusted and adjusted difference in FEV1 percent predicted between BLx and NHC by F508*del* status, with significantly lower FEV1 percent predicted in F508del heterozygous patients in the BLx/NHC group (β = -16.92, 95% CI = (-33.01, -0.82), *p* = 0.040). When adjusted for the potential confounding variables, the association for patients with 1 copy of F508*del* still trended toward significant (β = -15.57, 95% CI = (-32.31, 1.16), *p* = 0.068) but the results remained non-significant for the other two subgroups (without F508 mutation: β = -9.65, 95% CI = (-27.15, 7.84), *p* = 0.273; with 2 copies of F508 mutation: β = -0.67, 95% CI = (-17.43, 16.08), *p* = 0.937).

**Table 2:** Unadjusted and adjusted FEV1 percent predicted by genotype. #

	N,% * (BLx, NHC)	Delta FEV1 percent predicted unadjusted (CI)**	*p*	Delta FEV1 percent predicted adjusted (CI)	*p*
Heterozygous F508	9 (23%), 97 (43%)	− 16.92 (− 33.01, − 0.82)	0.040	− 15.57 (− 32.31, 1.16)	0.068
Homozygous F508	13 (38%), 59 (28%)	− 4.60 (− 18.77, 9.55)	0.520	− 0.67 (− 17.42, 16.08)	0.937
No F508 mutation	13 (38%), 49 (24%)	− 11.70 (− 27.21, 3.82)	0.137	− 9.65 (− 25.15, 7.84)	0.273

^*^Missing data in 22 patients

^**^Reflects difference in FEV1 percent predicted of BLx group compared with NHC

^#^ Patients’ gender, age, age at diagnosis, presence of diabetes, median household income based on zip code of residence, and insurance type (private/Medicare vs. public (Medicaid)) were included in the model to adjust for potential confounding

## Discussion

Disease severity in CF is a result of complex relationships among multiple factors along a patient’s lifespan. Our study finds that minorities with CF have significantly lower FEV1 percent predicted than non-minority patients, even when controlled for factors that are known to affect health outcomes in CF. Others have reported that lung function for Hispanic individuals with CF is lower starting at a very young age, though rate of decline in FEV1 is not different, suggesting that factors early in life may drive this trend [11]. In our patient cohort, this reduced pulmonary function is present despite the fact that minority patients are diagnosed at a similar age as non-minorities, with similar rates of diabetes mellitus and *Pseudomonas* infection, two prognostic indicators in CF. Approximately half of patients were diagnosed before age 2 years; most of the reported cohort consists of patients diagnosed after universal implementation of newborn screening for CF. Access to medical care appears to be similar as measured by the number of out-patient visits to the specialized CF clinic, number of hospitalizations, and number of courses of intravenous antibiotics over the year.

We did find that indicators of low socioeconomic status, including low median income by zip code and state insurance are significantly more prevalent in the BLx population than in the NHC population cared for at our clinic. Low SES is associated with worse disease outcomes for CF [8, 12]. When controlled for federal (Medicaid alone as well as Medicaid/Medicare combined) vs. private insurance and median household income, minority status still trended toward significantly lower lung function, suggesting that these socioeconomic factors are at least partly responsible for worse outcomes in BLx patients. Primary CF genotype does not appear to be the main cause of these disparities and in fact, our study shows a higher prevalence of F508*del* homozygous (associated with more severe disease in general CF population) in BLx compared with NHC, similar to what has been reported previously [13]. Our study findings, focused on patients receiving care in New York City, are similar to those that have been previously published in some other regions of the US [10–12]. It appears that as of yet undefined and unmeasured social and cultural factors, possibly including implicit bias, play a role in health disparities in CF. These considerations are important when considering interventions to improve the course of this nearly universally fatal disease. This is further highlighted by the recent introduction of CFTR modulator therapy with potential to prolong life.

A limitation of our study is that cross sectional analysis does not take into account factors that are present early in life that may impact disease trajectory. We combined Black and Hispanic patients in the category of minority even though these are distinct populations. Also we were not able to analyze adherence to standard CF therapies which could influence lung function. Modulator use was similar between the two groups. Highly effective modulator therapy (HEMT) for the majority of patients with CF was approved in October 2019 and likely does not impact our findings since use did not begin for most patients until very late 2019. Eligibility for recently approved HEMT is similar in both groups in our study.

Although there was no difference in number of visits to the CF clinic, there may be contributing individual factors such as adherence to treatment regimens, self-management culture, health literacy and English proficiency that affect outcomes. This datum confirms the importance of design of culturally appropriate preventative and management strategies to better understand how to direct interventions to this vulnerable CF population.

## Data Availability

Please contact author for data requests.
